# Case report: Exome sequencing revealed disease-causing variants in a patient with spondylospinal thoracic dysostosis

**DOI:** 10.3389/fped.2023.1132023

**Published:** 2023-09-07

**Authors:** Sami Bouchoucha, Asma Chikhaoui, Dorra Najjar, Khouloud Zayoud, Mohamed Zouari, Mohamed Nabil Nessib, Rym Kéfi, Houda Yacoub-Youssef

**Affiliations:** ^1^ Service Orthopédie, Hôpital D’enfant Béchir Hamza, Tunis, Tunisia; ^2^ Laboratoire de Génomique Biomédicale et Oncogénétique, LR16IPT05, Institut Pasteur de Tunis, Université Tunis El Manar, Tunis, Tunisia; ^3^ Genomics Platform, Institut Pasteur de Tunis, Université Tunis El Manar, Tunis, Tunisia

**Keywords:** whole exome sequencing, rare orthopedic disorders, *TPM2*, spondylospinal thoracic dysostosis, oligogenic inheritance

## Abstract

**Background:**

Spondylocostal dysostosis is a rare genetic disorder caused by mutations in *DLL3*, *MESP2*, *LFNG*, *HES7*, *TBX6*, and *RIPPLY2*. A particular form of this disorder characterized by the association of spondylocostal dysostosis with multiple pterygia has been reported and called spondylospinal thoracic dysostosis. Both disorders affect the spine and ribs, leading to abnormal development of the spine. Spondylospinal thoracic dysostosis is a rare syndrome characterized by the association of multiple vertebral segmentation defects, thoracic cage deformity, and multiple pterygia. This syndrome can be considered a different form of the described spondylocostal dysostosis. However, no genetic testing has been conducted for this rare disorder so far.

**Methods:**

We report here the case of an 18-month-old female patient presenting the clinical and radiological features of spondylospinal thoracic dysostosis. To determine the underlying genetic etiology, whole exome sequencing (WES) and Sanger sequencing were performed.

**Results:**

Using WES, we identified a variant in the *TPM2* gene c. 628C>T, already reported in the non-lethal form of multiple pterygium syndrome. In addition, following the analysis of WES data, using bioinformatic tools, for oligogenic diseases, we identified candidate modifier genes, *CAP2* and *ADCY6*, that could impact the clinical manifestations.

**Conclusion:**

We showed a potential association between *TPM2* and the uncommon spondylocostal dysostosis phenotype that would require further validation on larger cohort.

## 1. Introduction

Spondylocostal dysostosis (SCD) is a group of rare orthopedic disorders defined by multiple vertebral segmentation defects and rib deformities. Six genes are known to cause this disease, namely, *DLL3* (1), *MESP2* (2), *LFNG* (3), *HES7* (4), *TBX6* (5), and *RIPPLY2* (6). Recently, two other genes *DMRT2* and *SLC35A3* (7) have been associated with SCD-like form (8).

Affected individuals have a short trunk and a short neck with limited mobility. Spine deformity occurs in 10 contiguous vertebrae with mild and often non-progressive scoliosis. There is also a deformity of the ribs which can be fused, missing, or fanning out of the spine in a “crab-like” fashion. Respiratory complications are among the most common causes of morbidity and mortality in affected individuals. Deformity of the rib cage affects lung development, which causes lung infections and respiratory failure (9).

The association of SCD with fusion of the spinal processes of the vertebrae and multiple pterygium has been rarely reported and called spondylospinal thoracic dysostosis (SSTD) (10). SSTD seems to represent a separate clinical entity with overlapping findings with SCD. To date, no study reported cases of SSTD in North Africa or investigated the genetic etiology of this particular syndrome.

Several variations in distinct genes might occasionally result in a more severe phenotype that affects how the disease manifests itself in Mendelian disorders; this phenomenon is defined as oligogenic inheritance. Many Mendelian disorders, such as Gaucher’s disease, have been linked to oligogenic inheritance (11, 12).

In this work, we describe a patient with a complex phenotype suggestive of SSTD for whom whole exome sequencing (WES) revealed the presence of the pathogenic *TPM2* variant. We further analyzed WES data using bioinformatic tools for investigating the variant with oligogenic inheritance. Our findings point to a list of possible modifiers genes that might influence the patient's phenotype.

## 2. Materials and methods

### 2.1. Subject and ethical consent

This study was approved by the Institut Pasteur de Tunis (IPT) Biomedical Ethics Committee in Tunisia (reference 2021/10/E/V1), in accordance with the Declaration of Helsinki. A female patient affected by a complex phenotype suggestive of spondylocostal dysostoses syndrome was identified by the referral doctor. Written informed consent was obtained from both parents of the patient.

### 2.2. Whole exome sequencing

Genomic DNA from the affected individual and from his parents were extracted from peripheral blood. WES was performed via the Illumina NovaSeq 6000 platform. Raw paired-end reads were aligned using bwa to version hg38 of the reference genome (13). Picard and TrimGalore were used to identify duplicate reads. The GATK4 was then used to do variant detection. Finally, Annovar was used to annotate each variant (14). The VARAFT tool was used to further filter the variations.

### 2.3. *In silico* variant prioritization

By taking into account the subsequent filtering procedures on the VARAFT program (15), the list of candidate variants from WES was obtained. Only exonic, splicing, non-synonymous, and stop-gain variants with high coverage rate were taken into consideration. We removed variations that PolyPhen and SIFT predicted to be benign or tolerable and kept those with a CADD score of at least 15. Following this process, we were able to get a list of 28 potential variations, both homozygous and heterozygous, that was further explored for oligogenic inheritance via (ORVAL, Oligogenic Resource for Variant AnaLysis) (16). This strategy was adapted from a previous work on autism spectrum (17). We also prepared a file with genes related or possibly related to possible major phenotype according to human phenotype ontology (HPO) to be used as a “gene panel” file for variant filtering on ORVAL. This list was obtained using the following keywords: “myopathy,” “rib fusion,” “scoliosis,” and “pterygium”. From the output file, we kept only variants with a high pathogenicity score.

### 2.4. Sanger sequencing for WES data validation

The validation of *TPM2* variation from WES data was done using AmpliTaq Gold DNA Polymerase (Applied Biosystems, Foster City, CA, USA). Primers were selected from a previous study (18), F: CACAGTGGGAAGGTAGCAT/R: CGTAGGCTCCTGGTCATCT, under a Tm of 55°C. PCR products were sequenced with BigDye Terminator v3.1 (Applied Biosystems) on the ABI Prism 3500 sequencer (Applied Biosystems).

### 2.5. Network analysis of candidate genes

We employed the GeneCodis4 tool to reveal the enrichment of annotations of the identified genes and their link with the phenotype. All previous selected variants were further analyzed giving the fact that *TPM2* did not pass with other variants through ORVAL analysis (19). Among the available databases accessible via GeneCodis4, we focused on the HPO domain.

## 3. Results

### 3.1. Clinical investigation

#### 3.1.1. General description

**Figure 1:**
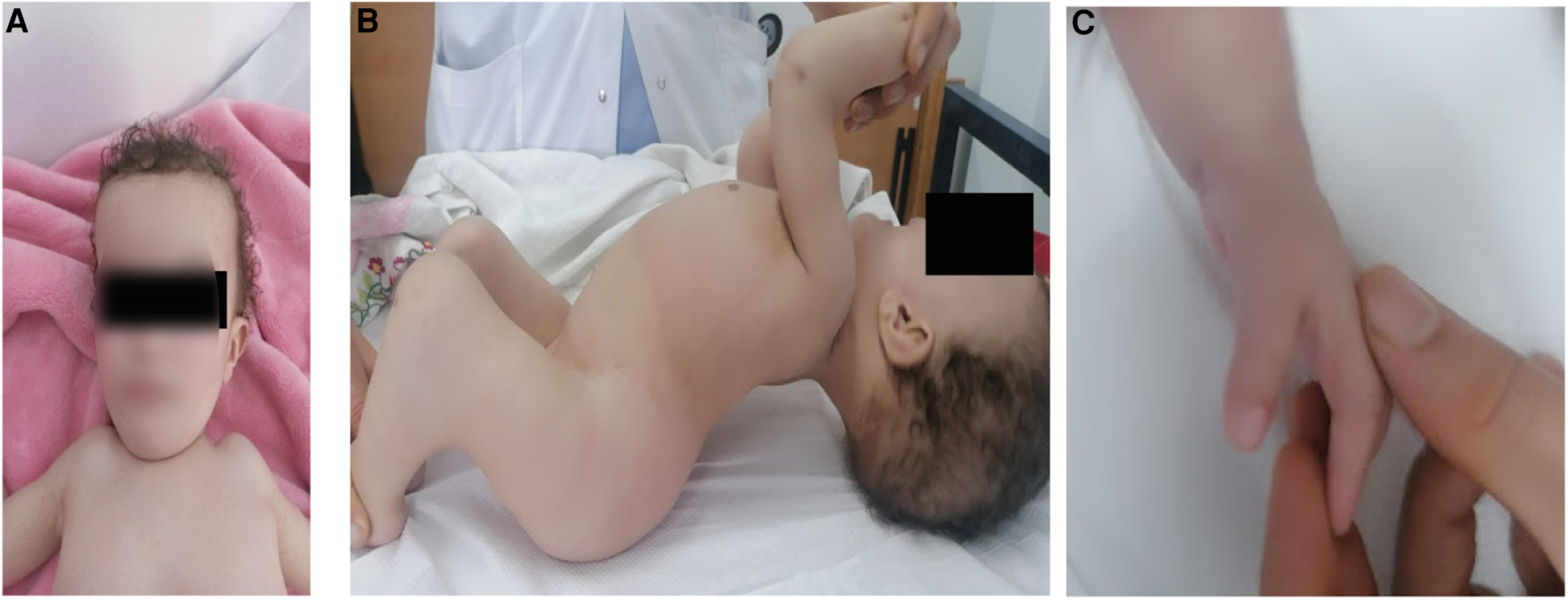
Clinical images of patient V-5 illustrating (**A**) broad forehead, (**B**) pterygium of the knee and hyperlordosis and (**C**) camptodactyly on the fingers.

The proband (patient V5) is an 18-month-old female, born to healthy parents from the North of Tunisia who were first-degree cousins. During the enquiry, the mother confirmed a normal pregnancy, normal movement of the fetus, and no signs of hypo/akinesia. Her birth weight was 2,500 g. She was of short stature with a height of 72 cm (<−4 SD) and had a short trunk and a short neck with limited mobility. She was neither able to sit nor stand owing to severe hyperlordosis of the entire spine and a severe flexion contracture of the knees. Head control was achieved at 5 months and eye contact was seen at the age of 10 months. The child had multiple joint contractures mainly at the elbows, fingers, and knees. She also had multiple pterygium, mainly in the inguinal region and the knees. There was camptodactyly on the fingers with both hands associated to pterygium. The patient also had a broad forehead with no other facial dysmorphism (Figure 1). No developmental and language delay were observed. In addition, no respiratory manifestations were noted upon examination. The clinical examination did not show any neurological disorder. There was no spasticity, muscle weakness, or a positive Babinski sign. It was not possible to examine the muscle stretch reflex due to extreme joint stiffness. Considering her young age, the patient did not undergo any surgery to correct bone deformities. At the age of 2.5 years, the parents informed us that the child died of respiratory failure following a lung infection.

After pedigree analysis, we found that the propositus had a deceased cousin (case V-1) who exhibited the same clinical symptoms. We also discovered other related cases in the same family (V-4, V-11, and V-12) who presented epilepsy (Supplementary Figure S1). Both parents were healthy and did not suffer from any bone or muscle abnormalities.

#### 3.1.2. Radiological findings

**Figure 2:**
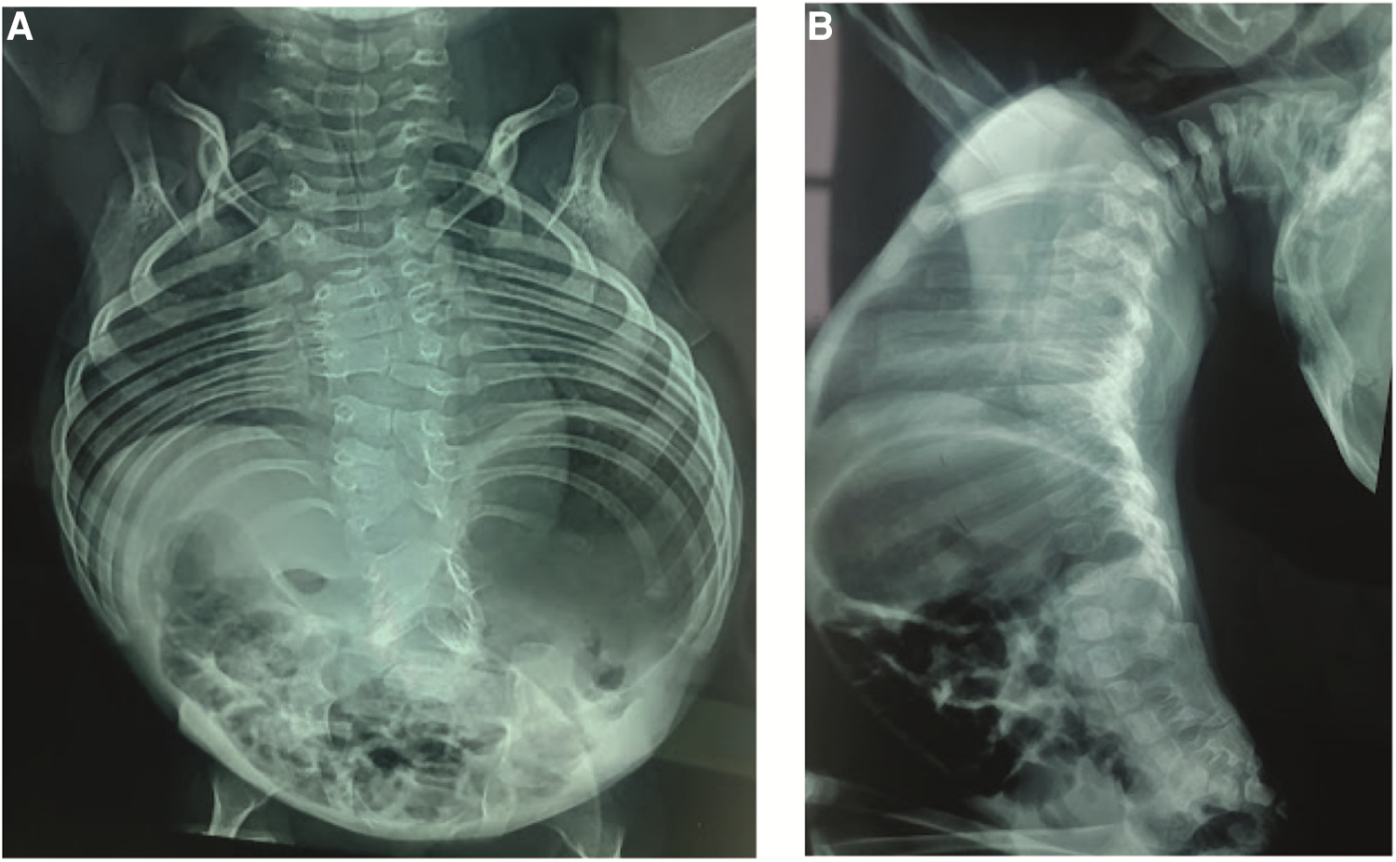
(**A**) Anteroposterior x-rays view showing the multiple vertebral defects and the “crab-like” aspect of the rib cage. (**B**) Lateral view showing the hyperlordosis and the fusion of the spinal processes.

Anteroposterior (AP) and lateral x-rays of the entire spine showed multiple vertebral segmentation defects with irregularly shaped vertebrae, bloc vertebrae, rachischisis, and multiple hemivertebrae defects affecting the whole spine with fusion of the spinal processes and a hyperlordosis that spans the entire spine. The ribs fan out from the vertebra in a “crab-like” fashion. There was no frontal deformity of the spine (Figure 2).

#### 3.1.3. Other clinical investigations

An electromyography (EMG) was performed and did not show any evidence of muscular pathology. The muscular biopsy did not show any sign of muscular disease as well. In details, hematoxylin–eosin staining and Gomori trichrome did not reveal nemaline bodies; moreover, ATPase and NADH staining did not show any particular anomalies.

### 3.2. WES genetic results

First assessment of WES data revealed the presence 98,237 homozygous and heterozygous variations that were subject to further filtering. A first investigation done on whole exome data showed no pathogenic variant in *DLL3*, *MESP2*, *LFNG*, *HES7*, *TBX6*, *RIPPLY2*, *DMRT2*, and *SLC35A3* genes that are known to be associated with SCD syndrome.

Further examination of rare variants in WES data that could be associated with the clinical features of the phenotype of the patient, such as the arthrogryposis and the multiple pterygium, did not reveal any variant in the acetylcholine receptor subunits (*CHRNG*, *CHRND*, *CHRNA1*) and other genes such as *MUSK*, *DOK7*, *RAPSN*, and *MYH3*. However, we found a homozygous variation in the *TPM2* gene that is located in exon 6 NM_001301226: c.628C>T: p.Gln210*. Variant segregation analyses using Sanger sequencing showed that the parents were heterozygous for this variant (Supplementary Figure S2).

#### 3.2.1. Possible implication of modifier genes

Giving the complexity of the patient's phenotype and that *TPM2* have been previously associated with other orthopedic disorders, we suspected the involvement of putative modifier genes that could be implicated in the clinical manifestations. We further filtered rare variants using ORVAL bioinformatic tool for oligogenic diseases; as a result, none of the selected variants were directly associated with *TPM2*. We then processed the related genes and their connections to each other and to the clinical manifestations via GeneCodis 4 tool to limit our selection to three genes (Table 1).

**Table 1:** A summary of the most significant variations discovered in the patient.

Gene	Chr	Variation	dbSNP ID	Depth	GnomAD frequency	ACMG criteria	ACMG classification and interpretation	MutationTaster_pred	Polyphen2_HDIV_pred	SIFT_pred	Variation type	Genotype
*CAP2*	6	NM_006366.3:c.877C>T (exon 6) p.Arg293Trp	rs201321988	108.0	0.00003978	PM2, PP3	Uncertain significance	D	D	D	Non-synonymous SNV	Hetrozygous
*SYNE2*	14	NM_015180:c.18304G>A (exon101):p.Glu6102Lys	rs560736067	111.0	0.00009473	PM2, PP3	Uncertain significance	D	D	D	Non-synonymous SNV	Hetrozygous
*ADCY6*	12	NM_015270:c.1640T>C (exon 8):p. Ile547Thr	rs143114060	76.0	0.003044	PP2. PP3, BP6	Uncertain significance	D	D	D	Non-synonymous SNV	Hetrozygous
*TPM2*	9	NM_003289.4:c.628C>T (exon6): p.Gln210*	rs199476154	72.0	Absent	PS3, PM2, PP3	Likely pathogenic	A			Stop-gain	Homozygous

SNV, single nucleotide variation.

**Figure 3:**
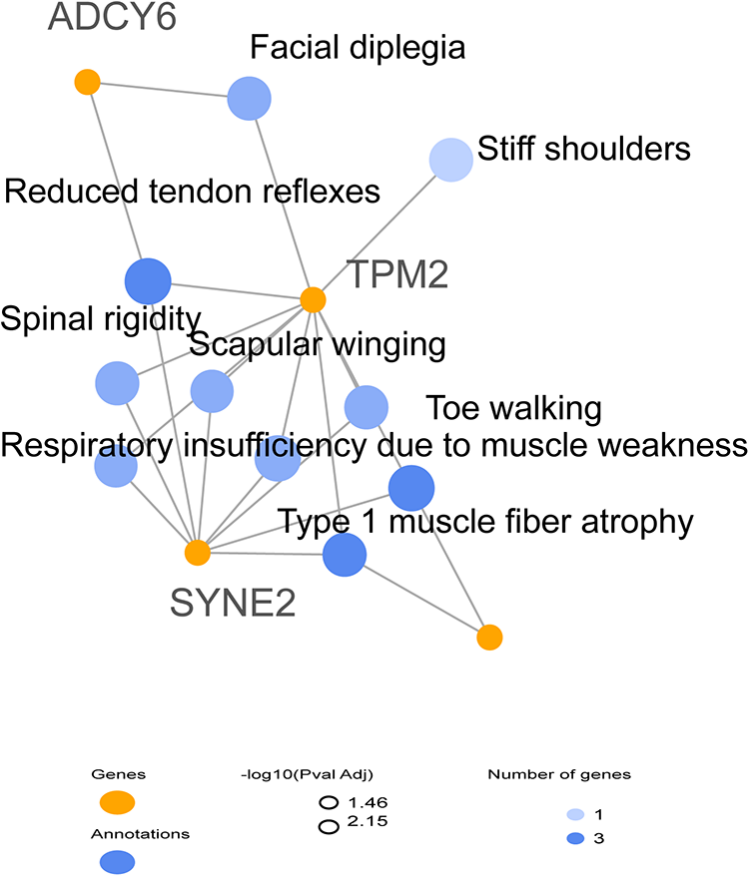
HPO network analysis of candidate modifier genes related to the patient phenotype suggesting the implication of *SYNE2*, *ADCY6*, and *CAP2* in clinical manifestations.

The results indicated that each of *SYNE2*, *ADCY6*, and *CAP2* genes seem to be implicated in the clinical manifestations such as muscle weakness, muscle atrophy, and reduced muscle reflexes by interacting with *TPM2* (Figure 3). We subsequently investigated the possible involvement of these variants in the regulatory process using Regulomedb (https://regulomedb.org/), which highlighted possible implication of variants in *ADCY6* and *CAP2*. Sanger sequencing revealed that *SYNE2* variants were inherited from the healthy mother, while in *CAP2*, it was a *de novo* variant found only in the patient. The variation in *ADCY6* was inherited from the healthy father (Results not shown).

## 4. Discussion

The clinical phenotype of the patient in our study consists of multiple deformities of the axial skeleton including a defect of segmentation of nearly all the vertebrae with multiple irregularly shaped vertebral bodies, bloc vertebrae, rachischisis, and multiple hemivertebrae. These deformities are associated with a severe lordosis of the entire spine with fusion of the spinous processes that impair the sitting ability of the child. Another axial anomaly is represented by the deformity of the rib cage with a fanning out of the ribs from the costovertebral junction in a “crab-like” fashion (20). These clinical features are highly consistent with the diagnosis of SCD (9). The latter are a heterogenous group of rare genetic disorders also known as spondylothoracic dysplasia, costovertebral dysplasia, or Jarcho–Levin syndrome transmitted on an autosomal-recessive or an autosomal-dominant way (21). These diseases are caused by mutation in *DLL3*, *MESP2*, *LFNG*, *HES7*, *TBX6*, *RIPPLY2*, *DMRT2*, and *SLC35A3* genes (21). The clinical and radiological distinction between the different types of SCD is based on differences in the vertebral and rib anomalies with different degrees of thoracic insufficiency that can be lethal in extreme cases (22). But none of the genetically identified types of SCD include multiple pterygia and arthrogryposis as it has been observed in our patient, which also cannot be explained by the multiple pterygium syndrome (MPS) because of the absence of akinesia and variants in the genes related to this disorder.

The association of vertebral segmentation defects and rib anomalies similar to what has been described in SCD, with fusion of the spinal processes and severe hyperlordosis of the entire spine and multiple pterygia with arthrogryposis, seems to be a separate entity. Johnson et al. termed this association SSTD stressing the fusion of the spinal processes and the resulted hyperlordosis (10). Becerra-Solano et al. named this particular entity the Turkel–Chen–Johnson syndrome after the first three authors who described the association of an SCD phenotype with extended fusion of the spinal processes, multiple pterygia, and arthrogryposis (23). To the best of our knowledge, only seven cases of SSTD have been reported previously (10, 23, 24). Table 2 compares the clinical and radiological manifestation found in SCD SSTD, and our patient. SSTD was considered lethal by most authors as the six first reported cases died at an early age due to thoracic insufficiency. This was also the case in our patient. However, Becerra-Solano et al. reported the case of a healthy 16-year-old Mexican girl, who goes to school without respiratory failure (23). As no genetic diagnosis for this association was known, the authors suggested to perform WES to sort out SSTD from SCD which gives very similar vertebral segmentation defects and rib anomalies. The genetic investigation carried out in this study suggests that Tropomyosin gene (*TPM*) mutations are associated with SSTD. Mutations in the tropomyosin-2 gene (*TPM2*) have been linked to different clinical manifestations ranging from pure distal arthrogryposis to cap disease and nemaline myopathy (NM) (25, 26), with more than 35 disease-causing mutations (26). The latter has been ruled out by histological analysis. Among these diseases, the non-lethal Escobar variant of MPS is characterized by multiple contractures of the joints (pterygia), webbing of the skin (syndactyly), and anomalies throughout the body and shows marked phenotypic variability (18, 27–29). Multiple pterygium, dwarfism, and facial dysmorphism are among the principal signs of the non-lethal form of MPS. Vertebral segmentation defects can also be seen but are usually localized and do not span the entire spine associated with rib fusions (30). Apart from SCD, the clinical phenotype of our patient is also consistent with the diagnosis of the Escobar syndrome although she lacked the characteristic facial features of the disease (31). In our study, the investigation of the genetic data obtained following whole exome sequencing and validation by Sanger sequencing identified the variation c.628 C>T in the *TPM2* gene that results in the emergence of a premature stop codon. This variation has already been reported in four Algerian patients with Escobar syndrome associated with NM (27). As in our patient, this was also reported in two other Tunisian patients with Escobar syndrome who did not exhibit myopathy (18). We therefore presume the absence of a clear phenotype–genotype correlation and the implication of other modifier genes in these complex syndromes as suggested by Najjar et al. (18). It is also important to note that since we used WES for genetic investigation, it is impossible to exclude other genetic factors responsible for the vertebral phenotype separately, in the setting of consanguinity that is known to be high in Tunisian population (32). As further analysis such as whole genome sequencing has not been performed, non-coding variants or del-dups in other genes have not been investigated but may be involved.

**Table 2:** Comparison of clinical and radiological manifestations in SSTD, SCD, and our case.

	SCD	SSTD(23)	Our patient
Multiple pterygia	−	+	+
Multiple segmentation defects	+	+	+
Crab-like aspect of the ribs	+	+	+
Extensive fusion of the spinous processes	−	+	+

The WES analysis also revealed mutations in the *SYNE2*, *ADCY6*, and *CAP2* genes that can be involved in the clinical manifestations in the patient. But none of the diseases caused by these mutations correspond to what was observed in our patient. American college of medical genetics and genomics (ACMG) classification and *in silico* prediction further delineate *SYNE2*, *ADCY6*, and *CAP2* as they display a potential role of modifier genes by playing a role of actin binding proteins (33–35). Indeed, we found a heterozygous variant in *SYNE2* gene, and this gene encodes for the Nesprin 2 protein, which is highly expressed in cardiac and skeletal muscles (36, 37). Previous studies suggest that mutations in this gene are associated with autosomal-dominant Emery–Dreifuss muscular dystrophy phenotypes (38–40). These myopathies are typically characterized by early contractures of the elbow flexors and Achilles tendons, spine rigidity, gradual progressive muscle wasting, weakness that begin at the humeroperoneal stage, and cardiopathy (39, 41). However, histological analysis of muscle biopsy and EMG revealed that this variant did not cause muscle pathology in our case. In addition, Sanger sequencing revealed that it was a heterozygous variant inherited from the healthy mother, with the lowest score with RegulomeDB and could be, therefore, the least implicated in the clinical manifestations of our patient.

The second variant that could play a role in the clinical particularities of this patient was observed also at a heterozygous state. Indeed, *CAP2*, which encodes the cyclase-associated protein (CAP), an enzyme that regulates the actin cytoskeleton, is critical for cell polarity and development (42). *CAP2* is mostly expressed in the heart, skeletal muscle, brain, and skin (43). It is related to autosomal-recessive dilated cardiomyopathy (44) and more recently to NM (45).

Through oligogenic analysis, we have also highlighted the possible involvement of a variant in *ADCY6* (Adenylate Cyclase 6) gene. This gene encodes for an enzyme from the adenylate cyclase family that is involved in the generation of cAMP implicated in myelinating signals in Schwann cells throughout development (46). It was previously associated with autosomal-recessive forms of arthrogryposis multiplex congenita (47). More recently, it has been also linked to recessive form of lethal congenital contracture syndrome 8, which consists of multiple joint contractures at birth, hypertonia, decreased tendon reflexes, and respiratory insufficiency (48). However, its presence in a heterozygous state as in our case could play a role in the particular clinical manifestation of our patient.

It is worth noting that the combination of the heterozygous variants in *CAP2*, *ADCY6*, and *SYNE2*, associated with the loss of function in *TPM2*, could also be related to cardiomyopathy. As both parents were apparently healthy and did not exhibit any clinical problems, we cannot, however, exclude any cardiac complication in the patient who died young during the time of investigation and did not undergo deep clinical examination. The same consideration applies for muscle atrophy, which was not observed following histological analysis, but cannot be ruled out due to her sudden death with a pulmonary infection.

SSTD is characterized by an overlap between SCD and Escobar syndrome. It is transmitted in an autosomal-recessive inheritance. In our patient, the sole mutation that can explain the clinical manifestations is the variant in the *TPM2* gene that we found. However, this mutation by itself cannot account for all of the disease variability giving the fact that it has been related to several syndromes (27). A contribution of other unknown modifier genes, environmental factors, or their combination is highly suspected (49). This study supports also the utility of the WES approach, particularly in patients who do not exhibit clear phenotypic feature related to one specific syndrome, and point out the need to do further investigations about the functionalities of *TPM2* in muscle and orthopedic disorders.

Through this report, we listed a possible variant that may be involved in disease severity based on the clinical manifestations observed in our case study. However, a larger cohort and further functional analysis could provide more information about gene interaction effects on the entire genome. As a result, many disease-related genetic connections remain yet to be discovered, and this report would provide elements of comparison for other similar clinical cases in order to define this syndrome with more precision.

Our findings could also raise concerns about the clinical concept of spondylospinal thoracic dysostosis as a separate entity disorder, as some of the known patients with SSTD may be cases of Escobar syndrome, clinically different due to genetic heterogeneity and due to the possible implication of modifier genes.

## Data Availability

The datasets presented in this article are not readily available because of ethical and privacy restrictions. Requests to access the datasets should be directed to the corresponding author.

## Supplementary material

Supplementary Figure 1 Pedigree of the affected family. Filled symbols denote affected patient with the same phenotype; proband is represented with an arrow; yellow filled symbols represent reported related individuals suffering from epilepsy.

Supplementary Figure 2 Electropherogram of the c.628 C>T mutation present at exon 6 of the *TPM2* gene detected by Sanger sequencing in patient V-5 and her parent.
