# Supplementary figures and images for: Case report: Exome sequencing revealed disease-causing variants in a patient with spondylospinal thoracic dysostosis
Source: Front Pediatr. 2023 Sep 7;11:1132023. doi: 10.3389/fped.2023.1132023 (PMC10512740; doi:10.3389/fped.2023.1132023)

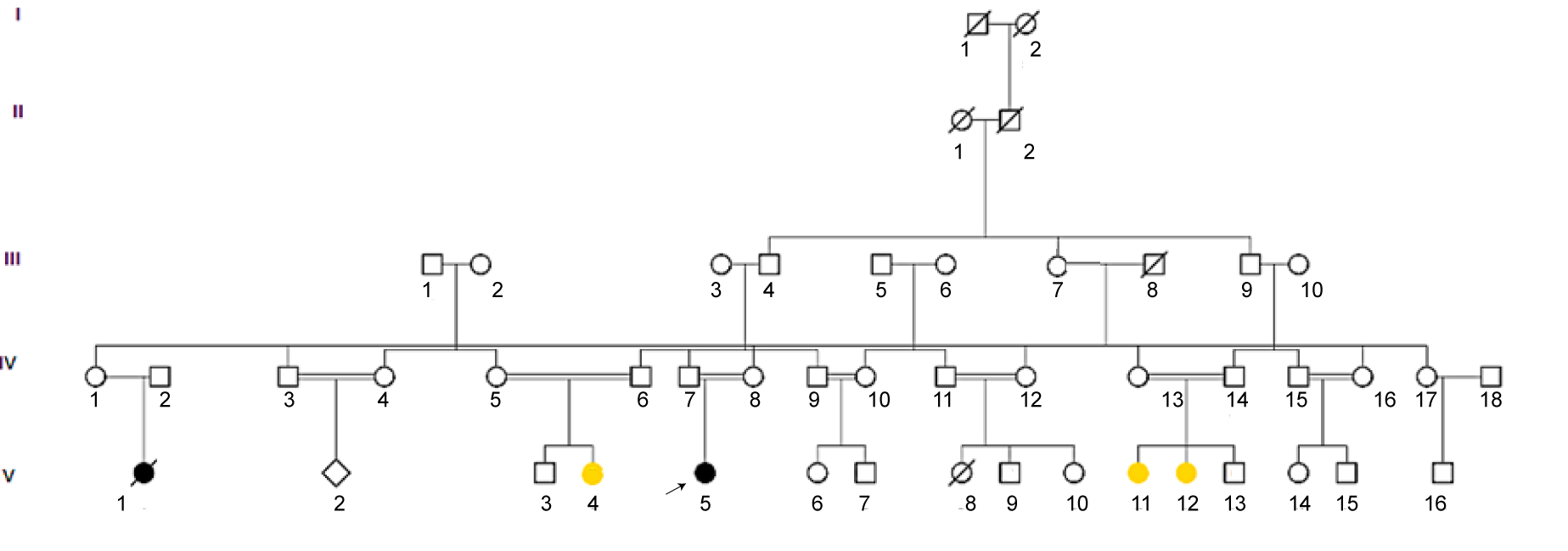

Supplement: Supplementary Figure 1 — Pedigree of the affected family. Filled symbols denote affected patient with the same phenotype; proband is represented with an arrow; yellow filled symbols represent reported related individuals suffering from epilepsy. [file Image1.tif]

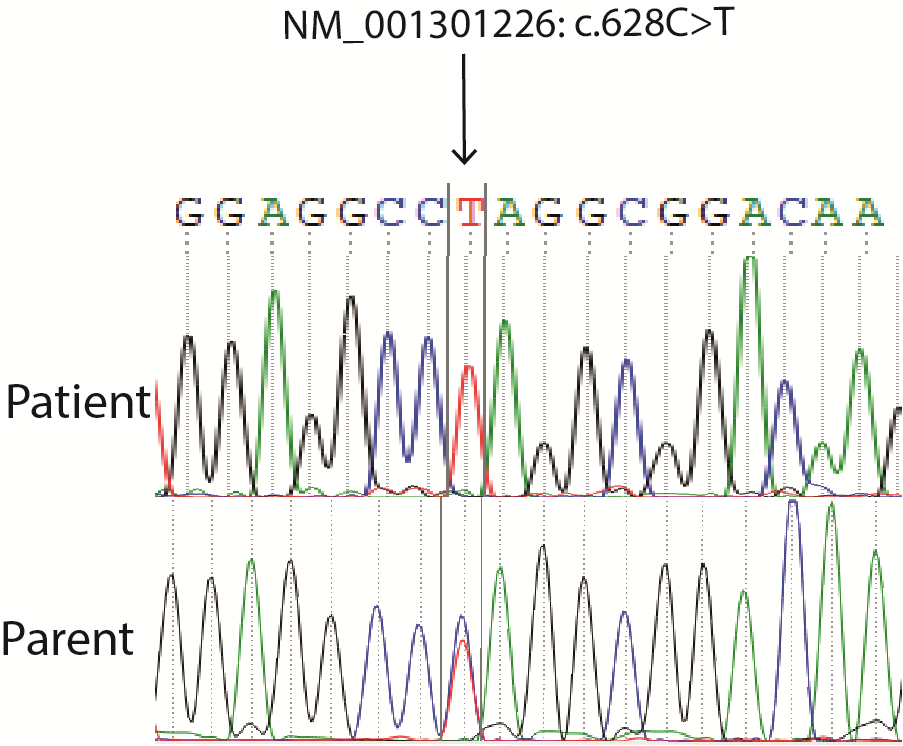

Supplement: Supplementary Figure 2 — Electropherogram of the c.628 C>T mutation present at exon 6 of the TPM2 gene detected by Sanger sequencing in patient V-5 and her parent. [file Image2.tif]
